# STAT3 expression is a prognostic marker in upper urinary tract urothelial carcinoma

**DOI:** 10.1371/journal.pone.0201256

**Published:** 2018-08-09

**Authors:** Kyosuke Matsuzaki, Kazutoshi Fujita, Yujiro Hayashi, Makoto Matsushita, Satoshi Nojima, Kentaro Jingushi, Taigo Kato, Atsunari Kawashima, Takeshi Ujike, Akira Nagahara, Motohide Uemura, Ryoichi Imamura, Seiji Yamaguchi, Hiroaki Fushimi, Hiroshi Miyamoto, Eiichi Morii, Norio Nonomura

**Affiliations:** 1 Department of Urology, Osaka University Graduate School of Medicine, Suita, Osaka, Japan; 2 Department of Pathology, Osaka University Graduate School of Medicine, Suita, Osaka, Japan; 3 Department of Therapeutic Urologic Oncology, Osaka University Graduate School of Medicine, Suita, Osaka, Japan; 4 Department of Urology, Osaka General Medical Center, Sumiyoshi-ku, Osaka, Japan; 5 Department of Pathology, Osaka General Medical Center, Sumiyoshi-ku, Osaka, Japan; 6 Departments of Pathology & Laboratory Medicine, Urology, and Oncology, University of Rochester Medical Center, Rochester, New York, United States of America; Duke University School of Medicine, UNITED STATES

## Abstract

Signal transducer and activator of transcription 3 (STAT3) plays a prominent role in the growth and invasion of several types of solid tumors. In this study, to assess the expression status and prognostic significance of the STAT3 pathway in upper urinary tract urothelial carcinoma (UTUC), we immunohistochemically stained for STAT3 and STAT3 pathway proteins, sphingosine-1-phosphate receptor 1 (S1PR1) and interleukin-6 (IL-6), in a tissue microarray containing 99 UTUC specimens. There were no significant associations between STAT3, S1PR1, or IL-6 expression pattern and tumor grade or pT stage. However, the patients with high STAT3 tumor had a significantly higher risk of both disease progression (p = 0.009) and cancer-specific mortality (p = 0.009), but not with tumors expressing S1PR1 or IL-6. High STAT3 expression in the nucleus was also associated with a significantly higher risk of both disease progression (p = 0.003) and cancer-specific mortality (p = 0.034). Multivariate analysis revealed that high STAT3 expression in the nucleus was significantly associated with cancer-specific survival after adjustment for pathological stage, lymph node involvement, lymphovascular invasion, and tumor grade (HR = 2.136, 95% CI = 1.009–4.767, p = 0.047). Our findings indicated that STAT3 could be a cancer-promoting factor and potentially a significant prognostic factor in UTUC.

## Introduction

Upper urinary tract urothelial carcinoma (UTUC) is relatively rare, accounting for approximately 5% to 10% of all urothelial tumors of the urinary tract [1–3]. Although urothelial carcinoma of the bladder and UTUC share many characteristics, practical, anatomical, biological, and molecular differences have been proven [4, 5]. Due to the lower incidence of UTUC compared with bladder cancer, little is known about the molecular markers confirmed to be useful for daily clinical decision making.

The signal transducer and activator of transcription (STAT) proteins are intracellular transcription factors that mediate various aspects of cellular immunity), proliferation, apoptosis, and differentiation) [6]. The STAT family includes seven members (STAT1, STAT2, STAT3, STAT4, STAT5A, STAT5B, and STAT6). Among them, STAT3 has been shown to play a prominent role in tumor growth and invasion [7]. In response to cytokines and growth factors, STAT3 is phosphorylated by receptor-associated Janus kinases (JAK), forms homo- or hetero-dimers, and translocates to the nucleus where it acts as a transcription activator [8].

S1PR1 is the one of the G-protein-coupled receptors for sphingosine-1-phosphate (S1P), a biologically active metabolite of sphingolipid [9]. S1P-S1PR1 signaling activates STAT3 [10]. IL-6 is a well-known traditional activator of STAT3 [11–13].

In this study, we evaluated the expression status of STAT3 pathway proteins, STAT3, S1PR1, and IL-6, in UTUC and analyzed their prognostic significance.

## Material and methods

### Patients and tissue samples

A UTUC tissue microarray (TMA) was constructed with spotted triplicate tumor samples from 99 patients who underwent radical nephroureterectomy performed with curative intent between 1997 and 2011 at Osaka General Medical Center, Osaka, Japan, as previously described [14–16]. Appropriate approval was obtained from the local institutional review board (Osaka General Medical Center Institutional Review Board, Protocol Number: 25–2014) before construction and use of the TMA, and written informed consent was obtained from all patients. Clinicopathological characteristics of the patients were obtained from medical records and follow-up data at the time of TMA production, and tissues samples were de-identified. Tumor progression was defined as the development of recurrence at the site of radical nephroureterectomy, lymph node metastasis, and/or visceral metastasis. Metachronous or synchronous lower tract recurrence (e.g., in the bladder) was not defined as tumor progression. Patients were followed up from initial diagnosis to the appearance of the event of interest or the end of the study. Patients who did not present the event of interest by the end of the study were censored from time-to-event analyses.

### Immunohistochemistry

Immunohistochemical staining was performed on 5-μm sections from the UTUC TMA using a primary antibody to STAT3 (LS-B4102, Cell Signaling Technology, Danvers, MA, USA), S1PR1 (sc-25489, Santa Cruz Biotechnology Inc., Santa Cruz, CA, USA) and IL-6 (ab-6672, Abcam, Cambridge, UK). We used normal kidney tissue as positive controls (S1 Fig). Sections were deparaffinized, rehydrated, and subjected to heat-induced antigen retrieval with a buffer solution using a steamer for 20 min before staining, and endogenous peroxidase activity was quenched with H_2_O_2_. Sections were then incubated with the appropriate primary antibody, and the DAKO EnVision™ Kit was used according to the manufacturer’s instructions. All of the stained sections were manually scored by two researchers (K.M. and K.F.) who were blinded to sample identity.

### Scoring system

TMA spots stained with each marker were evaluated for the pattern (nuclear and cytoplasmic), extent (percentage of positive cells), and intensity (0 to 3+ score) of staining. Cytoplasmic and nuclear staining was assessed for STAT3, and cytoplasmic staining was assessed for S1PR1 and IL-6 positivity. To account for the percentage of positive cells and staining intensity, an “H score” was assigned to each TMA spot as the sum of the products of the intensity (0, negative; 1, weakly positive; 2, moderately positive; and 3, strongly positive) and the extent of immunoexpression (0 to 100%), obtaining a value from 0 to 300, as previously described [14]. The final H score for each case was defined as the average score of triplicate TMA spots and was used during statistical analyses for all markers. For statistical analysis, the patients were divided into two groups according to the H score (High: H score > the median, Low: H score ≤ the median).

### Statistical analysis

Statistical analyses were performed using JMP^®^ Pro 13.2.0 (SAS Institute Inc., Cary, NC, USA). Patient characteristics were compared using the Mann-Whitney U test and χ^2^-test. The survival rates were determined using the Kaplan-Meier method and compared with the log-rank test. A Cox proportional hazards model was performed to determine the statistical significance of prognostic indicators in a multivariate setting. Differences were considered statistically significant when the p value was < 0.05.

## Results

Table 1 shows the characteristics of the 99 patients. The patients included 60 men and 39 women with a median (range) age of 71 (48–87) years at the time of surgery and a median (range) follow-up of 47 (2–173) months after surgery. Included in these patients were 45 renal pelvic tumors and 50 ureteral tumors (4 patients with tumors at both sites), 15 low-grade urothelial carcinomas and 84 high-grade urothelial carcinomas, 37 non-muscle-invasive tumors (pTa or pT1) and 62 muscle-invasive tumors (pT2, pT3, or pT4), 44 small tumors and 38 large tumors (17 patients without the record of tumor size), 84 pN0 tumors and 12 pN+ tumors (3 patients with pNx tumors), and two pM1 tumors. These two pM patients had peritoneal dissemination and distant lymph node metastases (merentery lymph nodes), and concomitant radical metastasectomy were performed. There were two patients with positive surgical margin, and one patient had pT2 and another had pT3 tumor. During follow-up, metachronous or synchronous recurrence in the lower urinary tract was observed in 32 patients. The details of the patients information were shown in S1 Table.

**Table 1:** Associations between clinicopathological profile of the patients and the expression level of STAT3, S1PR1 and IL-6.

Variable parameters	All cases	STAT3 expression			S1PR1 expression			IL-6 expression		
		Low N = 50	High N = 49	P-value	Low N = 50	High N = 49	P-value	Low N = 55	High N- = 44	P-value
**Age median (range)**	71 (48–87)	71.5 (51–87)	71 (48–84)	0.986	71 (52–87)	71 (48–85)	0.113	72 (53–87)	68 (48–85)	0.003
**Gender n, (%)**				0.592			0.485			0.027
male	60	29 (58.0%)	31 (63.3%)		32 (64.0%)	28 (57.1%)		28 (50.9%)	32 (72.7%)	
female	39	21 (42.0%)	18 (36.7%)		18 (36.0%)	21 (42.9%)		27 (49.1%)	12 (27.3%)	
**Tumor site n, (%)**				0.004			0.393			0.367
Renal pelvis	45	31 (62.0%)	14 (28.6%)		20 (40.0%)	25 (51.0%)		24 (43.6%)	21 (47.7%)	
Ureter	50	18 (36.0%)	32 (65.3%)		27 (54.0%)	23 (46.9%)		30 (54.6%)	20 (45.5%)	
Both	4	1 (2.0%)	3 (6.1%)		3 (6.0%)	1 (2.1%)		1 (1.8%)	3 (6.8%)	
**Tumor grade n, (%)**				0.425			0.149			0.851
Low grade	15	9 (18.0%)	6 (12.2%)		5 (10.0%)	10 (20.4%)		8 (14.5%)	7 (15.9%)	
High grade	84	41 (82.0%)	43 (87.8%)		45 (90.0%)	39 (79.6%)		47 (85.5%)	37 (84.1%)	
**Tumor size n, (%)**				0.557			0.193			0.706
<3cm	44	18 (36.0%)	26 (53.1%)		26 (52.0%)	18 (36.7%)		26 (47.3%)	18 (40.9%)	
≧3cm	38	18 (36.0%)	20 (40.8%)		17 (34.0%)	21 (42.9%)		24 (43.6%)	14 (31.8%)	
unknown	17	14 (28.0%)	3 (6.1%)		7 (14.0%)	10 (20.4%)		5 (9.1%)	12 (27.3%)	
**Pathologic stage n, (%)**				0.593			0.779			0.814
pTa	19	9 (18.0%)	10 (20.4%)		9 (18.0%)	10 (20.4%)		10 (18.2%)	9 (20.5%)	
pT1	18	12 (24.0%)	6 (12.2%)		10 (20.0%)	8 (16.3%)		10 (18.2%)	8 (18.2%)	
pT2	8	4 (8.0%)	4 (8.2%)		3 (6.0%)	5 (10.2%)		3 (5.5%)	5 (11.4%)	
pT3	48	23 (46.0%)	25 (51.0%)		26 (52.0%)	22 (44.9%)		28 (50.9%)	20 (45.5%)	
pT4	6	2 (4.0%)	4 (8.2%)		2 (4.0%)	4 (8.2%)		4 (7.3%)	2 (4.5%)	
**LVI n, (%)**				0.085			0.744			0.614
0	59	34 (68.0%)	25 (51.0%)		29 (58.0%)	30 (61.2%)		34 (61.8%)	25 (56.8%)	
1	40	16 (32.0%)	24 (49.0%)		21 (42.0%)	19 (38.8%)		21 (38.2%)	19 (43.2%)	
**Lymph node involvement n, (%)**				0.151			0.782			0.949
pN0	84	43 (86.0%)	41 (83.7%)		40 (80.0%)	44 (89.8%)		45 (81.8%)	39 (88.6%)	
pN1-3	12	4 (8.0%)	8 (16.3%)		7 (14.0%)	5 (10.2%)		7 (12.7%)	5 (11.4%)	
pNx	3	3 (6.0%)	0 (0%)		3 (6.0%)	0 (0%)		3 (5.5%)	0 (0%)	
**H score median (range)**		41.7 (0–63.4)	125 (66.7–265)		150 (0–225)	263.3 (226.7–300)		116.7 (93.3–150)	208.3 (153.3–300)	

**Fig 1:**
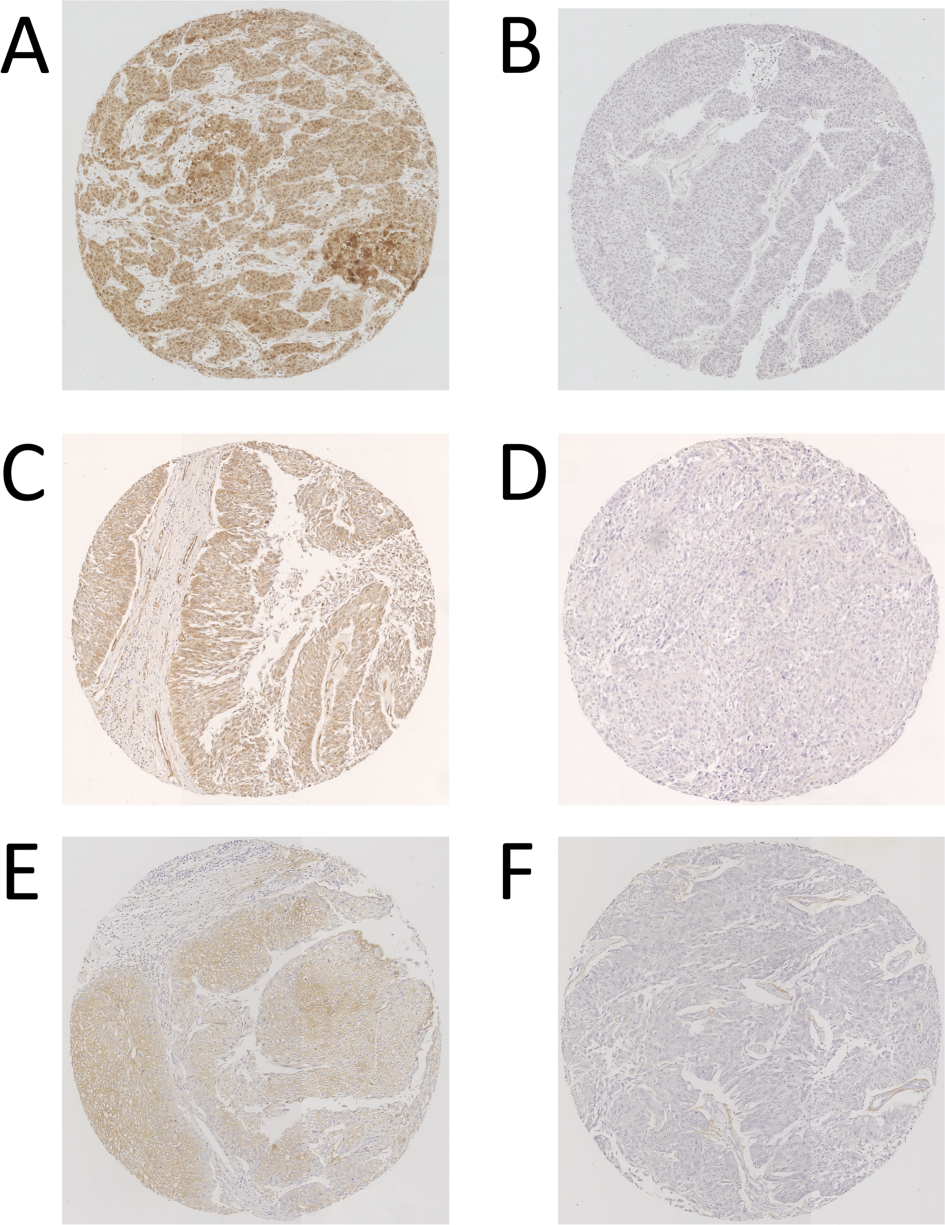
Typical patterns of immunohistochemical expression of STAT3 (A, High; B, Low), S1PR1 (C, High; D, Low), and IL-6 (E, High; F, Low) in UTUC tissue.

Representative patterns of immunoexpression are depicted in Fig 1. Table 1 also shows the association between the status of STAT3, S1PR1, or IL-6 expression in UTUC tissues and the clinicopathological profile. The ureteral UTUC showed significantly higher STAT3 expression (p = 0.004) than the UTUC of the renal pelvis, and men or younger-aged patients with UTUC had significantly higher IL-6 expression than women (p = 0.027) or older-aged patients with UTUC (p = 0.003). There was no significant difference in S1PR1 expression between the groups for various parameters. We analyzed the Spearman’s rank correlation coefficient of these 3 proteins expression, and it showed that S1PR1 expression weakly correlated with IL-6 expression (ρ = 0.302, p = 0.002). STAT3 expression did not correlated with both S1PR1 (ρ = 0.090, p = 0.375) and IL-6 (ρ = -0.154, p = 0.129). Because STAT3 activation has been reported to be related in chronic inflammation, we also evaluated the correlation of these markers with systemic inflammation. Neither of STAT3, S1PR1 and IL6 expression was associated with serum CRP, WBC and Neutrophil / Lymphocyte ratio (NLR) (S2 Table).

**Fig 2:**
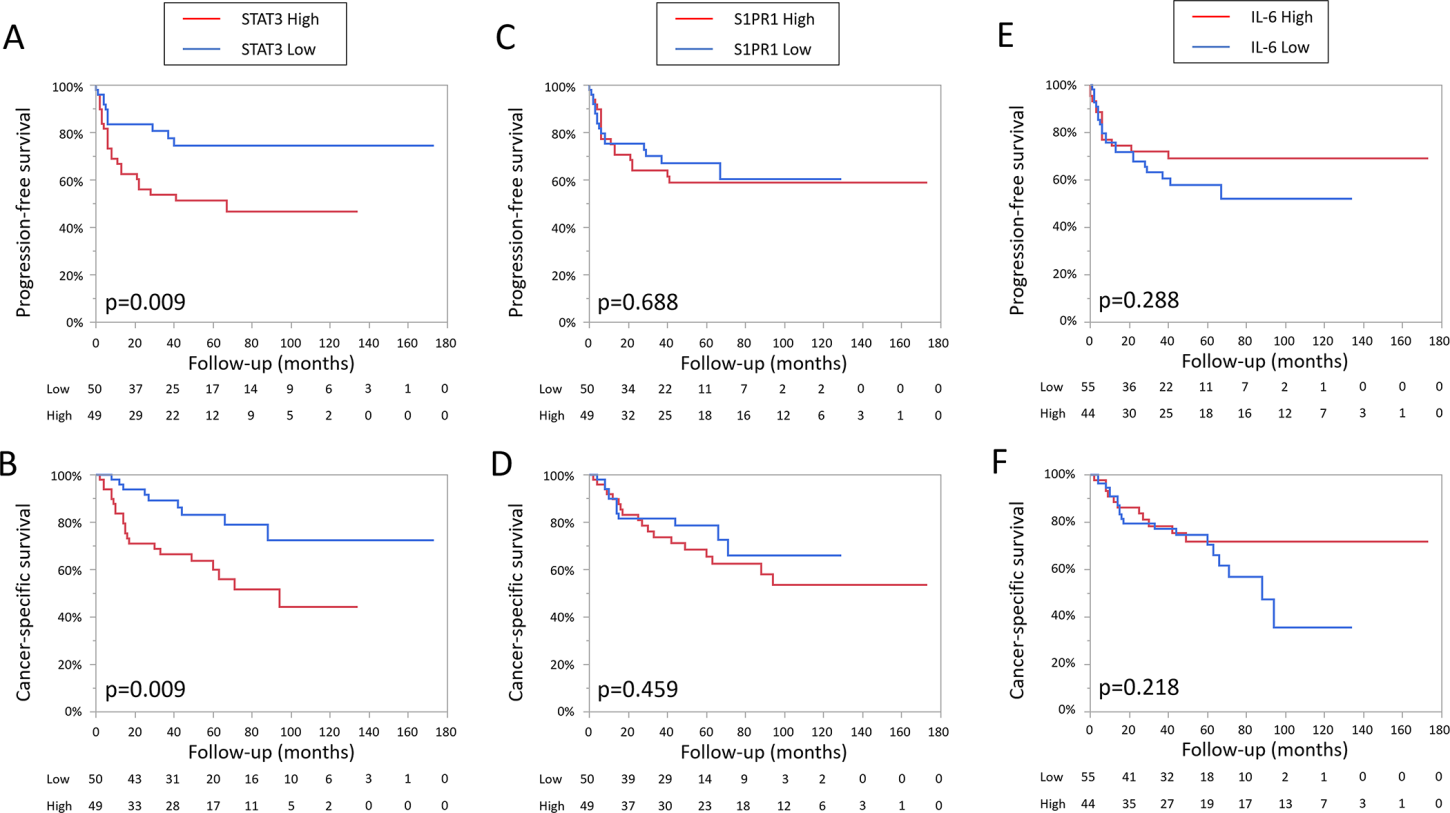
Progression-free survival and cancer-specific survival in patients with UTUC according to the expression levels of STAT3 (A, B), S1PR1 (C, D), and IL-6 (E, F).

Next, we performed Kaplan-Meier analysis coupled with the log-rank test to evaluate the prognostic values of STAT3, S1PR1, and IL-6 expression in UTUC. The patients with tumors showing high STAT3 expression had a significantly higher risk of both disease progression (p = 0.009) and cancer-specific mortality (p = 0.009), but not with tumors expressing S1PR1 or IL-6 (Fig 2). In subgroup of patients with high grade tumor (n = 84), the patients with high STAT3 tumor had a significantly higher risk of both disease progression (p = 0.008) and cancer-specific mortality (p = 0.008), but not with tumors expressing high S1PR1 (p = 0.257 and p = 0.161, respectively) or high IL-6 (p = 0.395 and p = 0.250, respectively). In the subgroup of low grade tumor, there was no significant difference in prognosis regardless of the expression level of STAT3, S1PR1 and IL6.

In patients with high stage tumor (≧pT3, n = 54), the patients with high STAT3 tumor had a significantly higher risk of both disease progression (p = 0.035) and cancer-specific mortality (p = 0.012), but not with tumors expressing high S1PR1 (p = 0.193 and p = 0.159, respectively) or high IL-6 (p = 0.609 and p = 0.365, respectively). In the subgroup of advanced tumor (≥pT1), only high STAT3 tumor had a significantly higher risk of both disease progression (p = 0.002) and cancer-specific mortality (p = 0.003). In the subgroup of non-invasive tumor (pTa) subgroup there was no significant difference regardless of the expression level of STAT3, S1PR1 and IL6.

Finally, we performed univariate logistic regression analysis of variable parameters associated with patient prognosis. It showed that the STAT3 score, nuclear STAT3 score, pathological stage (≥ pT3), lymph node involvement, lymphovascular invasion, and tumor grade were associated with both progression-free survival (Table 2) and cancer-specific survival (Table 3). Multivariate analysis with the Cox proportional hazards model revealed that pathological stage (≥ pT3), lymph node involvement, and tumor grade, but not STAT3 score, were significantly associated with progression-free survival and that pathological stage (≥ pT3) and tumor grade were significantly associated with cancer-specific survival (Tables 2 and 3, multivariate model 1). We also evaluated nuclear STAT3 expression by multivariate analysis, which revealed it to be significantly associated with cancer-specific survival after adjustment for pathological stage, lymph node involvement, lymphovascular invasion, and tumor grade (hazard ratio = 2.136, 95% confidence interval = 1.009–4.767, p = 0.047, Tables 2 and 3, multivariate model 2).

**Table 2:** Logistic regression analysis of variables associated with progression-free survival.

Variable	Progression-Free Survival								
	Univariate			Multivariate					
				Model 1			Model 2		
	HR	95% CI	P value	HR	95% CI	P value	HR	95% CI	P value
STAT3 score	2.473	1.240–5.257	0.010	1.309	0.341–1.625	0.491			
Nuclear STAT3 score	2.753	1.380–5.853	0.004				2.010	0.990–4.337	0.053
age	1.030	0.993–1.073	0.118						
≧pT3	13.471	4.797–56.228	<0.001	9.605	3.212–41.452	<0.001	9.891	3.321–42.627	<0.001
LVI	5.855	2.879–12.854	<0.001	1.818	0.782–4.416	0.166	1.936	0.859–4.567	0.111
pN stage	5.692	2.585–11.619	<0.001	3.439	1.448–7.909	0.006	3.300	1.396–7.535	0.008
Tumor grade	8.222	1.770–146.235	0.003	9.668	1.929–176.738	0.002	9.538	1.990–171.488	0.002
Tumor size	1.891	0.921–3.976	0.083						
Location (ureter vs pelvis)	1.062	0.535–2.139	0.863						

**Table 3:** Logistic regression analysis of variables associated with cancer-specific survival.

Variable	Cancer-Specific Survival								
	Univariate			Multivariate					
				Model 1			Model 2		
	HR	95% CI	P value	HR	95% CI	P value	HR	95% CI	P value
STAT3 score	2.725	1.285–6.272	0.008	1.891	0.863–4.491	0.113			
Nuclear STAT3 score	2.186	1.056–4.753	0.035				2.136	1.009–4.767	0.047
age	1.031	0.990–1.077	0.147						
≧pT3	36.061	7.683–643.242	<0.001	23.382	4.551–428.839	<0.001	23.387	4.618–427.152	<0.001
LVI	6.621	2.981–16.726	<0.001	1.893	0.762–5.191	0.173	2.309	0.949–6.217	0.066
pN stage	3.269	1.362–7.080	0.010	1.202	0.481–2.784	0.680	1.090	0.428–2.567	0.849
Tumor grade	7.569	1.614–134.996	0.005	5.865	1.194–106.061	0.025	8.061	1.691–144.539	0.004
Tumor size	1.366	0.619–3.043	0.437						
Location (ureter vs pelvis)	1.194	0.574–2.531	0.634						

We also performed logistic regression analysis in high grade or advanced (≥pT1) tumor subgroup (S3 and S4 Tables). In the subgroup of high grade tumor, STAT3 and nuclear STAT3 were significantly associated with both progression-free survival and cancer-specific survival in univariate analysis but not in multivariate analysis. In the subgroup of advanced (≥pT1) tumor, STAT3 and pSTAT3 were significantly associated with both progression-free survival and cancer-specific survival in univariate analysis. In multivariate analysis there was a trend toward significance in STAT3 (p = 0.090) and nuclear STAT3 (p = 0.079) for cancer-specific survival but not for progression-free survival.

## Discussion

The relation between inflammation and cancer progression has been well established. STAT3 is the major inflammation-promoting transcription factor shown to play important roles in cancer progression in various types of tumors [7, 8]. Although several studies have reported STAT3 as an important factor in the development of bladder cancer [17], the relation between STAT3 and UTUC progression remains unclear. In this study, we evaluated the relation between the expression of STAT3 pathway proteins, STAT3, S1PR1, and IL-6, and the prognosis of UTUC. There were no significant differences in the expression levels of STAT3 pathway proteins between low-grade versus high-grade UTUCs or non-muscle-invasive versus muscle-invasive UTUCs. However, our study showed that high expression of STAT3 was associated with poor prognosis, indicating that STAT3 was a cancer-promoting factor in UTUC.

**Fig 3:**
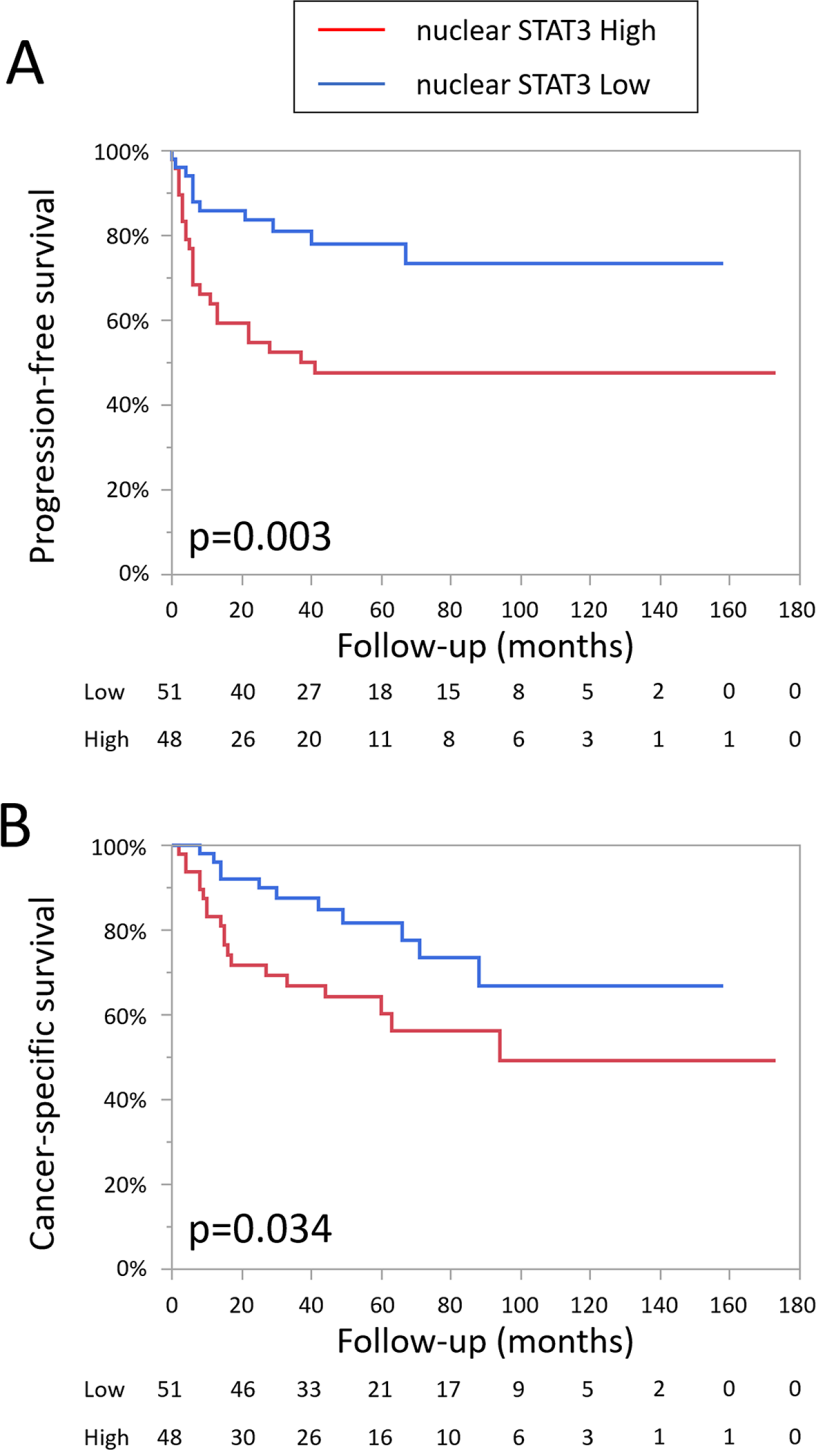
Progression-free survival (A) and cancer-specific survival (B) in patients with UTUC according to the expression level of STAT3 in the nucleus.

STAT3 is phosphorylated by upstream signals and translocates to the nucleus in which it acts as a transcription activator [8]. We also evaluated phopho-Stat3 (pSTAT3) activation. However there was no difference in prognosis between high pSTAT3 and low pSTAT3 (data not shown). This may be due to the condition of the formalin fixation. The time to the fixation could have been varied in each cases. Since phosphorylation of protein is not stable, pSTAT3 might have been affected by these conditions. We alternately evaluated STAT3 expression in the nucleus and found that the patients with high STAT3 expression in the tumor nucleus were at significantly higher risk of both disease progression (p = 0.003) and cancer-specific mortality than the other patients (p = 0.034, Fig 3).

We also focused on two proteins, S1PR1 and IL-6, upstream of the STAT3 pathway. S1PR1 expression in non-muscle-invasive bladder cancer was reported to be associated with poor prognosis [18]. IL-6 is the most well-known traditional activator of STAT3 [10–12], and positive staining of IL-6 in bladder cancer was reported to be significantly correlated with higher clinical stage and higher recurrence rate [19]. Although our study showed no association between the expression of S1PR1 or IL-6 and UTUC progression, this might be due to differences in the epidemiologic molecular and clinical characteristics between UTUC and bladder cancer. Another reason could be that STAT3 is regulated by many other factors such as cytokines, G-protein-coupled receptors, and Toll-like receptors [8].

Because STAT3 activation by the NFkB-IL-6 signaling pathway has been reported to be one of the mechanisms of carcinogenesis in chronic inflammation, we evaluated the correlation of these markers with systemic inflammation. In result, neither of STAT3, S1PR1 and IL6 expression was correlated with serum CRP, WBC and neutrophil / lymphocyte ratio (NLR). (S1 Table). However, these markers may be correlated with cancer-related immunity (Tumor-associated macrophages or regulatory T cells, etc), and further studies will be necessary to evaluate it.

Several studies reported the prognostic factors of various tissue-based molecular markers that are related to cell adhesion (metalloproteinase-9, E-cadherin, ParvB, Snail, b-catenin), cell signaling (EGFR, EMP3, HER2, PI3K/AKT, IGFBP, mTOR), angiogenesis (hypoxia-inducible factor-1), cell proliferation (Ki67, p27,cyclin D, NF-κB, Aurora-A), cell transport (GRP78), apoptosis (bcl-2, survivin) [20], immune response (NFATc1 [21]), and transcription factor (GATA3 [22]). Our study was the first report to show STAT3 as the prognostic factor of UTUC.

Recently it has been suggested that UTUC can be divided into four molecular subtypes with distinct clinical behaviors [23]. Although STAT3 was not associated with tumor stage nor tumor grade, STAT3 expression was the significant prognostic factor. For these reasons, we speculated that STAT3 expression might be associated with some molecular subtypes of urothelial carcinoma with worse prognosis such as basal-like type.

This study has a limitation. Because we used TMA samples instead of whole tumor sections, the staining heterogeneity could affect the evaluation of the expression levels. However, several studies have shown that multiple TMA spots adequately represent the expression of an entire section in the assessment of immunohistochemical markers [24].

## Conclusion

STAT3 expression was significantly associated with UTUC progression, and nuclear expression of STAT3 was the significant prognostic factor of UTUC-specific survival. Our study indicated that STAT3 could potentially be a new therapeutic target, but further studies will be necessary to fully determine its biological significance in the development and progression of UTUC.

## Supporting information

S1 Fig Positive controls of immunohistochemistry of STAT3, S1PR1 and IL-6 (normal kidney tissue). (TIF)

S1 Table The details of the patients information. (XLSX)

S2 Table Correlation coefficient (ρ) of STAT3, S1PR1 and IL6 with serum CRP, WBC and NLR. (DOCX)

S3 Table Logistic regression analysis of variables associated with progression-free survival (A) and cancer-specific survival (B) in high grade tumor subgroup.
(DOCX)

S4 Table **Logistic regression analysis of variables associated with progression-free survival (A) and cancer-specific survival** (B) **in advanced (≥pT1) tumor subgroup.**
(DOCX)
