# Supplementary figures and images for: STAT3 expression is a prognostic marker in upper urinary tract urothelial carcinoma
Source: PLoS One. 2018 Aug 9;13(8):e0201256. doi: 10.1371/journal.pone.0201256 (PMC6084864; doi:10.1371/journal.pone.0201256)

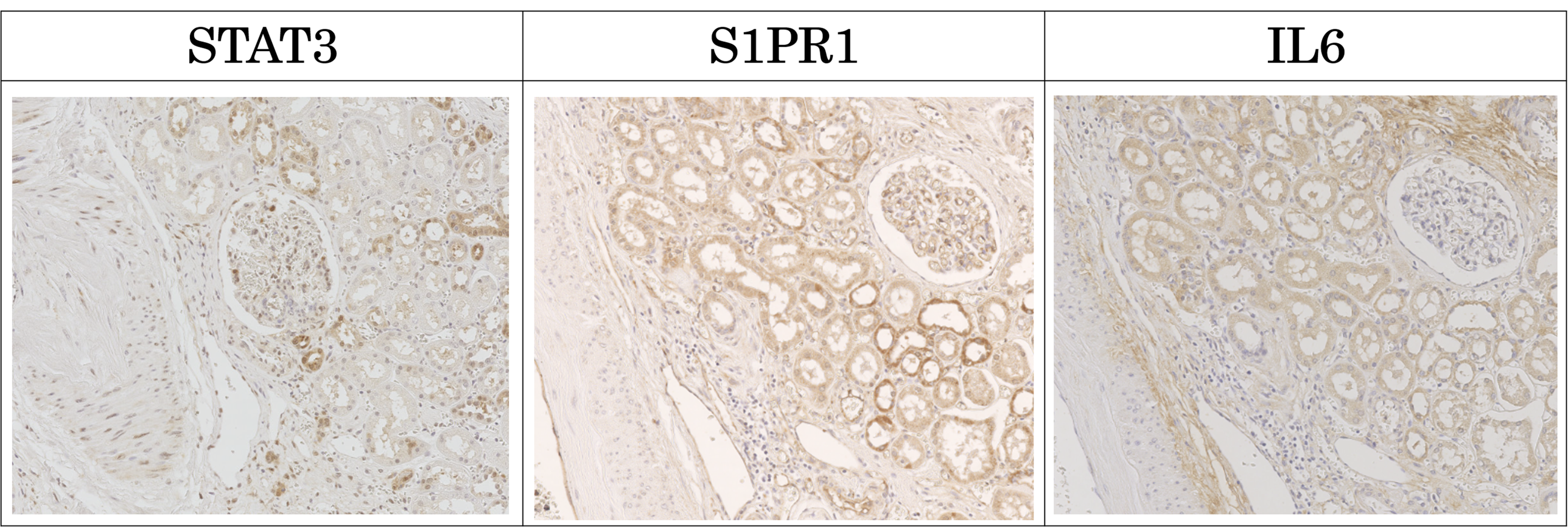

Supplement: S1 Fig — (TIF) [file pone.0201256.s001.tif]
