# Supplementary material for: STAT3 expression is a prognostic marker in upper urinary tract urothelial carcinoma
Source: PLoS One. 2018 Aug 9;13(8):e0201256. doi: 10.1371/journal.pone.0201256 (PMC6084864; doi:10.1371/journal.pone.0201256)
Supplement: S2 Table — (DOCX) [file pone.0201256.s003.docx]

**S1 Table: Correlation coefficient (ρ) of STAT3, S1PR1 and IL6 with serum CRP, WBC and NLR.**

|  | *CRP* | *WBC* | *NLR* |
| --- | --- | --- | --- |
| *STAT3* | *0.035* | *-0.035* | *-0.071* |
| *S1PR1* | *0.025* | *-0.041* | *-0.110* |
| *IL6* | *-0.343* | *-0.216* | *-0.251* |
