# Supplementary material for: STAT3 expression is a prognostic marker in upper urinary tract urothelial carcinoma
Source: PLoS One. 2018 Aug 9;13(8):e0201256. doi: 10.1371/journal.pone.0201256 (PMC6084864; doi:10.1371/journal.pone.0201256)
Supplement: S3 Table — Logistic regression analysis of variables associated with progression-free survival (A) and cancer-specific survival (B) in high grade tumor subgroup. (DOCX) [file pone.0201256.s004.docx]

**S2A Table. Logistic regression analysis of variables associated with progression-free survival in high grade tumor subgroup.**

| Variable | Progression-Free Survival | | | | | | | | |
| --- | --- | --- | --- | --- | --- | --- | --- | --- | --- |
|  | Univariate | | | Multivariate | | | | | |
|  |  |  |  | Model 1 | | | Model 2 | | |
|  | HR | 95% CI | P value | HR | 95% CI | P value | HR | 95% CI | P value |
| STAT3 score | 2.572 | 1.265-5.640 | 0.008 | 1.242 | 0.569-2.902 | 0.595 |  |  |  |
| Nuclear STAT3 score | 2.753 | 1.380-5.853 | 0.004 |  |  |  | 1.268 | 0.551-3.002 | 0.578 |
| age | 1.026 | 0.985-1.071 | 0.409 |  |  |  |  |  |  |
| ≧pT3 | 13.378 | 4.737-56.008 | <0.001 | 11.431 | 3.729-50.121 | <0.001 | 11.323 | 3.712-49.530 | <0.001 |
| LVI | 5.300 | 2.588-11.692 | <0.001 | 1.419 | 0.583-3.580 | 0.443 | 1.559 | 0.677-3.738 | 0.298 |
| pN stage | 6.322 | 2.838-13.110 | <0.001 | 5.249 | 2.017-13.532 | 0.001 | 5.054 | 1.933-13.208 | 0.001 |
| Tumor size | 2.174 | 1.042-4.680 | 0.038 | 1.776 | 0.791-4.028 | 0.163 | 1.596 | 0.675-3.881 | 0.287 |
| Location (ureter vs pelvis) | 1.034 | 0.515-2.119 | 0.515 |  |  |  |  |  |  |

**S2B Table. Logistic regression analysis of variables associated with cancer-specific survival in high grade tumor subgroup.**

| Variable | Cancer-Specific Survival | | | | | | | | |
| --- | --- | --- | --- | --- | --- | --- | --- | --- | --- |
|  | Univariate | | | Multivariate | | | | | |
|  |  |  |  | Model 1 | | | Model 2 | | |
|  | HR | 95% CI | P value | HR | 95% CI | P value | HR | 95% CI | P value |
| STAT3 score | 2.848 | 1.309-6.851 | 0.008 | 2.029 | 0.892-5.113 | 0.094 |  |  |  |
| Nuclear STAT3 score | 2.138 | 1.023-4.679 | 0.043 |  |  |  | 2.174 | 0.942-5.241 | 0.069 |
| age | 1.030 | 0.985-1.079 | 0.203 |  |  |  |  |  |  |
| ≧pT3 | 35.191 | 7.458-628.764 | <0.001 | 23.072 | 4.473-423.758 | <0.001 | 23.382 | 4.605-427.408 | <0.001 |
| LVI | 5.786 | 2.589-14.669 | <0.001 | 1.683 | 0.652-4.740 | 0.287 | 2.174 | 0.863-6.002 | 0.101 |
| pN stage | 3.189 | 1.324-6.947 | 0.012 | 1.254 | 0.447-3.413 | 0.659 | 1.276 | 0.456-3.452 | 0.634 |
| Tumor size | 1.488 | 0.663-3.401 | 0.334 | 1.067 | 0.418-2.697 | 0.890 | 0.772 | 0.286-2.065 | 0.603 |
| Location (ureter vs pelvis) | 1.185 | 0.561-2.569 | 0.658 |  |  |  |  |  |  |
