# Supplementary material for: STAT3 expression is a prognostic marker in upper urinary tract urothelial carcinoma
Source: PLoS One. 2018 Aug 9;13(8):e0201256. doi: 10.1371/journal.pone.0201256 (PMC6084864; doi:10.1371/journal.pone.0201256)
Supplement: S4 Table — Logistic regression analysis of variables associated with progression-free survival (A) and cancer-specific survival (B) in advanced (≥pT1) tumor subgroup. (DOCX) [file pone.0201256.s005.docx]

**S3A Table. Logistic regression analysis of variables associated with progression-free survival in advanced (≥pT1) tumor subgroup.**

| Variable | Progression-Free Survival | | | | | | | | |
| --- | --- | --- | --- | --- | --- | --- | --- | --- | --- |
|  | Univariate | | | Multivariate | | | | | |
|  |  |  |  | Model 1 | | | Model 2 | | |
|  | HR | 95% CI | P value | HR | 95% CI | P value | HR | 95% CI | P value |
| STAT3 score | 3.021 | 1.484-6.631 | 0.002 | 1.598 | 0.735-3.688 | 0.241 |  |  |  |
| Nuclear STAT3 score | 2.648 | 1.317-5.657 | 0.006 |  |  |  | 1.816 | 0.877-3.969 | 0.110 |
| age | 1.020 | 0.981-1.063 | 0.329 |  |  |  |  |  |  |
| LVI | 4.234 | 2.038-9.637 | <0.001 | 2.570 | 1.101-6.002 | 0.026 | 2.694 | 1.187-6.467 | 0.018 |
| pN stage | 4.472 | 2.027-9.162 | 0.001 | 2.490 | 1.061-5.631 | 0.037 | 2.442 | 1.045-5.488 | 0.040 |
| Tumor grade | 8.362 | 1.795-148.847 | 0.003 | 6.629 | 1.359-119.714 | 0.014 | 6.974 | 1.472-124.789 | 0.009 |
| Tumor size | 1.923 | 0.923-4.081 | 0.171 |  |  |  |  |  |  |
| Location (ureter vs pelvis) | 0.990 | 0.493-2.028 | 0.977 |  |  |  |  |  |  |

**S3B Table. Logistic regression analysis of variables associated with cancer-specific survival in advanced (≥pT1) tumor subgroup.**

| Variable | Cancer-Specific Survival | | | | | | | | |
| --- | --- | --- | --- | --- | --- | --- | --- | --- | --- |
|  | Univariate | | | Multivariate | | | | | |
|  |  |  |  | Model 1 | | | Model 2 | | |
|  | HR | 95% CI | P value | HR | 95% CI | P value | HR | 95% CI | P value |
| STAT3 score | 3.118 | 1.468-7.191 | 0.003 | 1.971 | 0.903-4.658 | 0.090 |  |  |  |
| Nuclear STAT3 score | 2.251 | 1.087-4.901 | 0.029 |  |  |  | 1.952 | 0.927-4.311 | 0.079 |
| age | 1.025 | 0.983-1.073 | 0.251 |  |  |  |  |  |  |
| LVI | 4.350 | 1.958-10.988 | <0.001 | 3.174 | 1.319-8.418 | 0.010 | 3.554 | 1.439-8.781 | 0.004 |
| pN stage | 2.478 | 1.032-5.367 | 0.043 | 1.175 | 0.470-2..726 | 0.626 | 1.133 | 0.449-2.653 | 0.781 |
| Tumor grade | 8.025 | 1.708-143.191 | 0.004 | 5.316 | 1.086-96.003 | 0.037 | 6.728 | 1.420-120.361 | 0.011 |
| Tumor size | 1.408 | 0.637-3.140 | 0.394 |  |  |  |  |  |  |
| Location (ureter vs pelvis) | 1.083 | 0.520-2.296 | 0.832 |  |  |  |  |  |  |
